# Endocytic Pathways Involved in Filovirus Entry: Advances, Implications and Future Directions

**DOI:** 10.3390/v4123647

**Published:** 2012-12-11

**Authors:** Suchita Bhattacharyya, Nirupama Mulherkar, Kartik Chandran

**Affiliations:** 1 Department of Atomic Energy-Centre for Excellence in Basic Sciences, University of Mumbai, Health Centre Building, Vidyanagari, Kalina, Santacruz East, Mumbai 400098, India; E-Mail: bsuchita@gmail.com; 2 Department of Microbiology and Immunology, Albert Einstein College of Medicine, 1300 Morris Park Ave, Bronx, NY 10461, USA; E-Mail: nirupamamulherkar@gmail.com

**Keywords:** filoviruses, viral entry, viral internalization, endocytosis, endocytic pathways, clathrin-mediated endocytosis, macropinocytosis, caveolae-mediated endocytosis

## Abstract

Detailed knowledge of the host-virus interactions that accompany filovirus entry into cells is expected to identify determinants of viral virulence and host range, and to yield targets for the development of antiviral therapeutics. While it is generally agreed that filovirus entry into the host cytoplasm requires viral internalization into acidic endosomal compartments and proteolytic cleavage of the envelope glycoprotein by endo/lysosomal cysteine proteases, our understanding of the specific endocytic pathways co-opted by filoviruses remains limited. This review addresses the current knowledge on cellular endocytic pathways implicated in filovirus entry, highlights the consensus as well as controversies, and discusses important remaining questions.

## 1. Architecture of Filovirus Virions and the Viral Glycoprotein, GP

Members of the family Filoviridae (filoviruses) are non-segmented negative-strand RNA viruses that produce filamentous enveloped virions. Filoviruses belong to one of three serologically, biochemically and genetically distinct genera—Ebolavirus, Marburgvirus, and “Cuevavirus” (tentative) [1,2]. Three ebolaviruses (Bundibugyo virus [BDBV], Ebola virus [EBOV], Sudan virus [SUDV]) and one marburgvirus (Marburg virus [MARV]) are associated with outbreaks of highly lethal hemorrhagic fever for which no approved vaccines and treatments are available (see [3,4] for recent reviews).

Filovirus virions are filamentous particles with a uniform diameter (~90 nm) but somewhat variable length (900–1,000 nm) and pleomorphic overall appearance [5,6]. These virions contain a single virus‑encoded membrane glycoprotein, GP, which is organized into homotrimeric spikes on the viral surface (see [7,8,9] for recent reviews). GP is necessary and sufficient to mediate viral entry into target cells. 

EBOV GP is encoded by two overlapping open-reading frames (ORF). The default polypeptide product of the GP gene is sGP, a small secreted glycoprotein of unknown function [10]. Expression of the full-length GP precursor requires the insertion of a non-templated adenosine residue by transcriptional RNA editing [11]. By contrast, MARV GP is encoded by a single ORF, and no sGP equivalent is produced [12]. The GP precursor is post-translationally cleaved by the pro-protein convertase furin within the Golgi compartment of virus-producer cells, yielding two disulfide-linked subunits, GP1 and GP2 [13]. The membrane-distal GP1 subunit binds to cellular receptors and controls the conformation of the GP2 transmembrane subunit. GP2 catalyzes fusion between viral and cellular membranes. GP1 contains a highly conserved N-terminal receptor-binding sequence and more variable C-terminal sequences, including an extensively glycosylated mucin-like domain. The GP2 subunit comprises an N-terminal internal fusion loop, N-terminal and C-terminal heptad repeat sequences whose refolding drives membrane fusion (see below), a transmembrane domain, and a short cytoplasmic tail (see [7,8,9] for recent reviews). 

## 2. Role of Acid pH and Endo/Lysosomal Host Factors in Filovirus Entry

Filoviruses are known to enter host cells via acid pH-dependent endocytic pathways [14,15,16]. Viral particles containing EBOV GP were shown to require Rab7 for entry [17] and co-localize with Rab7-positive endosomes following viral internalization [18,19]. MARV is also targeted to lysosomes after being endocytosed [16]. Within one or more endo/lysosomal compartments, host endosomal cysteine proteases (cysteine cathepsins) cleave GP1 to remove its variable C-terminal sequences, generating an entry intermediate comprising an N-terminal GP1 fragment and GP2 [20,21,22,23]. Recent work indicates that a cleaved form of GP must then engage Niemann-Pick C1 (NPC1), an endo/lysosomal cholesterol transporter that serves as a critical intracellular receptor for filovirus entry [19,24,25]. Additional undefined events downstream of GP-NPC1 binding are proposed to trigger the induction of GP conformational changes, including insertion of the GP2 fusion loop into the host membrane, and GP2 refolding into a ‘six-helix bundle’ configuration in which the C-terminal heptad repeats pack against grooves in a trimeric α-helical coiled-coil formed by the N-terminal heptad repeats [26,27,28,29,30]. These GP rearrangements are proposed to drive membrane merger and release of the viral nucleocapsid core into the cytoplasm. In addition to GP1 proteolytic cleavage [20], multiple steps in the GP2-mediated viral membrane fusion reaction may require endo/lysosomal acid pH [31,32,33,34]. Therefore, current findings indicate that filovirus particles must traffic to, and possibly enter the cytoplasm from, late endosomal and/or lysosomal compartments. 

The remainder of this article will focus on the upstream endocytic trafficking pathways that deliver viruses in general, and filoviruses in particular, to the intracellular sites where viral membrane fusion or penetration can occur.

## 3. Cellular Endocytic Pathways Implicated in Viral Entry

Cellular endosomes are pleomorphic structures, which fuse with one another for cargo trafficking. Cellular signaling pathways, as well as signals on the internalized receptors regulate sorting and trafficking of viruses after internalization. The major cellular endocytic pathways involved in entry of viruses into target cells include clathrin-mediated endocytosis, uptake by caveolae, macropinocytosis and phagocytosis. Several clathrin- and caveolae-independent endocytic pathways have also been reported [35,36], but they are not completely understood.

### 3.1. Clathrin-Mediated Endocytosis

Clathrin-mediated endocytosis is the most widely studied endocytic pathway and is used by several viruses for entry into target cells [37,38,39]. Viral entry via clathrin-mediated endocytosis involves the localization of viruses in clathrin-coated pits, which invaginate and are pinched off by dynamin. The pits then form endocytic vesicles, which fuse with early endosomes and travel further into the cytoplasm. Clathrin dissociates from these vesicles and remains in the cytoplasm until it is recruited to line newly formed pits.

Several cellular factors participate in and regulate various steps of the clathrin-mediated endocytic pathway. These include the coat protein clathrin, which assembles into a polyhedral lattice on the inner surface of the plasma membrane to form the coated pit. The PICALM protein promotes the assembly of clathrin triskelia into cages [40]. The HIP1 protein localizes with clathrin at the plasma membrane and is involved in the formation of the coated vesicle [41,42]. HIP1 can also bind to the adaptor protein AP-2 [43] (see below). LDLRAP1, which interacts with the cytoplasmic tail of the low density lipoprotein (LDL) receptor, can bind to both clathrin and AP-2 [44]. 

The adaptor proteins involved in the clathrin pathway initiate vesicle formation by bringing cargo molecules to the clathrin coat [45,46]. These include AP-2, which links the clathrin lattice to the cell membrane [47]; β-arrestins, which can bind to clathrin directly [48]; DAB2, which can sort the LDL receptor independently of AP-2 and LDLRAP1 [49]; and Eps15, which constitutively associates with AP-2 during clathrin-mediated endocytosis [37]. Interestingly, anthrax toxin is known to enter cells via an Eps15-, AP-2- and DAB2-independent clathrin pathway that requires AP-1 and β-arrestin [40]. 

In addition to Eps15, several other proteins are known to possess Eps homology (EH) domains such as Epsin 1, Intersectin 1 (ITSN1), REPS1 and REPS2 [50,51,52]. Epsin 1 is involved in clathrin‑mediated endocytosis of influenza A virus [53], whose entry was previously reported to be AP-2 independent [54]. Epsin 1 binds to both ITSN1 [55] and Eps15 [53] through the same EH‑domain binding sequence and therefore, simultaneous binding of Eps15 and ITSN1 is unlikely, suggesting that Epsin 1 and ITSN1 may act as alternate adaptors to substitute for the respective functions of AP-2 and Eps15 in the clathrin pathway. The EH domains of ITSN bind to epsin [55], while the Src homology 3 (SH3) domains bind to dynamin and synaptojanin [56]. Another adaptor protein NUMB, can bind to the α-adaptin subunit of AP-2 [57] and Eps15 [58]. 

Some of the regulatory proteins involved in the clathrin pathway include INPPL1, which recruits ITSN1 to clathrin-coated pits on the plasma membrane [59]. RalBP1/RLIP76, a Rac/Cdc42 guanine‑nucleotide activating protein, targets the AP-2 complex and regulates endocytosis [60]. REPS1 and REPS2 proteins associate with RalBP1 and act as molecular switches to coordinate the actions of the RalBP1-interacting Ral-GTPases [61]. Also, REPS1 forms complexes with two adaptor proteins Crk and Grb2 [61], while REPS2 can bind directly to epsin through its EH domain [62]. 

The scission of the clathrin-coated pits is carried out by the GTPase dynamin 2 (DYN-2), which assembles at the neck of the coated pits and functions as a constrictase to pinch off the pits [63]. BAR domain proteins such as amphiphysin and SNX9 are recruited to the sites of clathrin assembly where they induce membrane curvature, interact with actin and synaptojanin, and promote the recruitment of dynamin [64,65]. 

Taken together, these studies demonstrate that numerous cellular factors with overlapping functions participate in and regulate the clathrin pathway. Given the complexity of this pathway and the variations in the composition of individual clathrin pits, it would be interesting to examine how different viruses dictate the recruitment of specific adaptors and regulators to a clathrin pit.

### 3.2. Caveolae-Mediated Endocytosis

Caveolae-mediated endocytosis involves the formation of small flask-shaped invaginations of the plasma membrane [66]. Unlike the clathrin pathway, caveolae-mediated endocytosis is usually not a constitutive process and predominantly occurs upon cell stimulation [67,68].

Caveolae are a specialized form of lipid rafts primarily composed of cholesterol and sphingolipids [69]. The shape and structure of caveolae is determined by caveolin, a protein that binds cholesterol and self associates to form a striated coat on the surface of the invaginations [70]. The cavin proteins are known to regulate caveolae structure [71]. PTRF-cavin binds to caveolin and stabilizes the membrane curvature to produce the characteristic flask shape of caveolae [69].

Several regulatory factors are known to be involved in various steps of the caveolae pathway. These include Cdc42 and RhoA, which facilitate caveolae formation by promoting actin polymerization and binding to caveolin 1 [72]; tyrosine kinases and phosphatases, which trigger downstream signaling pathways after cargo binding; integrins, which regulate trafficking of caveolae; and protein kinase C (PKC), which stimulates uptake by caveolae [73,74].

Dynamin can directly bind to caveolin [75] and is suggested to act as a scission factor by triggering fission of the caveolae [76]. Intersectin 2 localizes at the neck of the caveolae and regulates the activity of dynamin. An adaptor protein, NOSTRIN, recruits dynamin to the caveolae [69].

Phosphorylation of caveolin 1 by Src leads to caveolar internalization. The cytoskeletal components associated with the caveolae pathway include actin, filamin and microtubules, which facilitate formation as well as internalization of caveolae [69,74].

Upon internalization, caveolae form characteristic grape-like, multi-caveolar complexes of heterogeneous morphology known as caveosomes. Rab 5 regulates the fusion of caveosomes with early endosomes. The endosomal route taken by different ligands internalized by the caveolae pathway is regulated by various Rabs, kinases and phosphatases [69].

Viruses such as SV40 enter through caveolae [77] which are approximately 60 nm in size [66]. Since filoviruses have also been reported to enter via caveolae-mediated endocytosis [78,79], it is conceivable that caveolae can adapt to fit the size of their cargo.

Several sterol-binding chemicals such as methyl-β-cyclodextrin and filipin can block caveolae‑mediated endocytosis [80,81] but their inhibitory effects are not restricted to the caveolar pathway alone [82]. 

### 3.3. Macropinocytosis

Macropinocytosis is a transient actin-dependent endocytic process that is typically employed for cellular uptake of fluids and large solutes via large (0.5–10 µm diameter) irregular-shaped vacuoles or macropinosomes. 

Macropinocytosis is generally initiated by external stimuli (growth factor-mediated) resulting in the formation of actin-driven cellular protrusions called membrane ruffles that can fuse to form macropinosomes. Rho family GTPases (Rac1, Cdc42) and p21-activated kinase (Pak1) are known important mediators of ruffle formation in addition to Na^+^ influx and H^+^ efflux. Membrane ruffles also contain several regulators of actin polymerization, disassembly, stabilization and cytoskeletal membrane attachment such as Arp2/3, VASP, WAVE, PKC and several classes of myosins, all of which are known to play a role in ruffle extension and macropinosome formation [83,84]. 

Several adaptors and regulators have been reported to be involved in macropinocytosis. These include the adaptor complex-1 (AP-1), which is required for macropinosome formation [85], Abi1 [86], TBC1D3 [87], c-Cbl [88] and NHE1 which is required to achieve the necessary H^+^ concentration to promote actin polymerization during macropinocytosis [89]. Other cellular factors involved in this pathway include epidermal growth factor (EGF) receptor, phosphatidylinositol (PI) 3‑kinase (PI3K), Phospholipase C (PLC), the ARF-family of GTPases, and CtBP1 (carboxyl-terminus binding protein 1), which is associated with macropinosome closure [83]. 

Several viruses are known to enter via macropinocytosis and distinct cellular factors have been shown to be capable of inducing macropinocytosis of these virus particles. For example, human adenovirus serotype 3 requires alpha v integrins [90], vaccinia virus uses phosphatidyl serine exposed on its surface to induce its uptake via macropinocytosis [91,92], Kaposi's sarcoma-associated herpesvirus requires the adaptor protein c-Cbl and myosin IIA [88,93], coxsackievirus requires occludin and Rab34 [94] and Nipah virus induces macropinocytosis via a signaling cascade involving Rac1 and Cdc42 [95]. 

Several chemical agents such as dimethyl amiloride [96], cytochalasin D and PI3K inhibitors [97] have been shown to block macropinocytosis but they are not specific inhibitors of this pathway [82].

### 3.4. Phagocytosis

Phagocytosis is a receptor-mediated form of endocytosis that includes a number of closely related yet distinct mechanisms. It is carried out by specialized cells such as neutrophils, monocytes and macrophages and is typically used by cells to clear large pathogens and debris. Like macropinocytosis, phagocytosis is also associated with actin-dependence, large vacuole size and cellular factors such as RhoA, Cdc42, Rac-1 and PI3K. However, unlike macropinocytosis, phagocytosis involves cargo‑specific receptor interactions resulting in a signaling cascade that triggers cytoskeletal rearrangements. This causes formation of cell surface extensions that specifically zipper up around the cargo and form a cargo-sized vacuole called the phagosome [66]. Dynamin-2 has been reported to be required for the closure of phagosomes whereas macropinosome closure is associated with CtBP1 [98].

Several adaptor proteins are involved in phagocytosis, which include Syk, Grb2, Gab2, and CrkII. Lipids are also actively involved in phagocytosis and anionic phospholipids such as phosphatidyl serine and phosphoinositides are known to make the inner leaflet of the plasma membrane negatively charged during the early steps of this process [99]. Other cellular factors that are recruited to phagosomes include PI3K, Phospholipase D, IQGAP1, amphiphysin1 and adhesion proteins [74].

Herpes simplex virus 1 [100] and foot-and-mouth disease virus [79] are known to enter cells via phagocytosis. PI3K inhibitors [97] and filamentous actin depolymerizing agents can block phagocytosis but their inhibitory effects are not restricted to this pathway [82].

### 3.5. Clathrin- and Caveolae-Independent Endocytic Pathways

Several clathrin- and caveolae-independent endocytic pathways involving internalization of ligands in non-coated vesicles have been reported, including two distinct pathways of lipid transport to the Golgi apparatus [101]. The IL-2 receptor is suggested to be a marker for these pathways [102], whose entry requires Rac1, Paks, and cortactin [103].

Each of these clathrin- and caveolae-independent pathways uses distinct cellular factors for internalization. Feline infectious peritonitis virus was shown to enter via a dynamin-dependent pathway [104]. Dynamin is also implicated in the entry of coxsackievirus A9 through a pathway that requires β2-microglobulin and Arf6 [105]. By contrast, lymphocytic choriomeningitis virus (LCMV) entry requires cholesterol but is independent of dynamin, Arf6 and actin [106,107]. Similarly, human papillomavirus type 16 entry is also independent of dynamin and Rho GTPases but requires tetraspanin-enriched microdomains, PI3K, PKC, and actin [108] 

In addition to these clathrin- and caveolae-independent pathways that are known to be utilized by different viruses for entry, several other pathways exist that are not currently associated with viral entry. These include the GEEC pathway involved in endocytosis of GPI-anchored proteins [109], the flotillin-1-dependent pathway utilized by GPI-anchored proteins and proteoglycans [110], the Arf6‑dependent pathway used by MHC antigens [111] and the IL-2 pathway, which is utilized for internalization of cytokine receptors [74]. 

Thus, there are many distinct clathrin- and caveolae-independent pathways that have been partially characterized, and perhaps several more that are yet to be uncovered. It would be interesting to explore if any of these additional pathways are utilized by viruses for entry. 

## 4. Cellular Endocytic Pathways Implicated in Filovirus Entry

The involvement of microtubules, lipid rafts and membrane cholesterol in EBOV entry was reported by several groups [112,113,114]. Caveolae, which are composed of lipid microdomains, were also implicated in filovirus entry [78] but this finding was later disputed [115]. However, a more recent report has again proposed the involvement of caveolae in filovirus entry [79]. Hence, the role of caveolae in filovirus entry remains unclear.

Both clathrin-mediated endocytosis [116,117,118] and macropinocytosis [17,18,118,119] were shown to be involved in filovirus entry by several groups. Additionally, some studies have suggested that filoviruses concurrently use multiple endocytic pathways for entry, including clathrin- and caveolae‑mediated endocytosis [78]; clathrin, caveolae and macropinocytic pathways [79]; and macropinocytosis and clathrin-mediated endocytosis [118]. It is currently unclear how the use of distinct internalization pathways by filoviruses is influenced by viral isolate, cell/tissue type, and type of surrogate viral particle employed (native filovirus virions, filamentous virus-like particles (VLPs), or retrovirus or VSV pseudotypes).

### 4.1. Role of Clathrin Endocytic Pathway in Filovirus Entry

The average size of a clathrin-coated pit is approximately 120 nm. However Listeria monocytogenes, which is usually 2 μm in length, has been shown to enter target cells using the clathrin pathway [120]. More recently, Cureton and co-workers showed that bullet-shaped VSV particles (~200 nm in length) are internalized through vesicles containing partial clathrin coats, and whose formation requires actin polymerization in a cargo size-dependent manner [121,122]. These findings led to the premise that clathrin-coated pits adapt to accommodate the size of their cargo and could therefore play a role in filovirus entry. 

A study with chemical inhibitors proposed that wild type filoviruses could enter Vero cells by clathrin as well as caveolar pathways [123]. However, chemical agents such as chlorpromazine and sucrose, which are known to prevent recycling of clathrin to the plasma membrane [124], do not specifically block the clathrin pathway and can also inhibit other endocytic pathways [82]. Therefore, data obtained using chemical inhibitors must be substantiated with other approaches to establish the specificity of target inhibition. 

Using multiple approaches to inhibit several cellular factors involved in the clathrin pathway, Bhattacharyya and co-workers showed that retrovirus pseudotypes containing EBOV GP utilized clathrin-mediated endocytosis to enter several cell lines, including human endothelial cells [117]. A more exhaustive follow-up study revealed a differential requirement for several key cellular components of the clathrin pathway in cell entry by retrovirus pseudotypes bearing MARV GP *versus* EBOV GP. Importantly, EBOV GP-mediated entry required Eps15, AP-2 and DAB2, whereas MARV GP-mediated entry was independent of these cellular factors and instead requires the adaptor protein β‑arrestin-1 [116] Subsequent studies have also corroborated the involvement of clathrin-mediated endocytosis in EBOV entry in a human glioblastoma cell line using feline immunodeficiency virus pseudotyped with EBOV GP [79], and in HeLa cells using EBOV GP VLPs [118]. 

The capacity of different types of particles containing filovirus glycoproteins to use clathrin‑mediated endocytosis for cell entry raises the possibility that the filamentous morphology of native filovirus virions is not a critical determinant of the route of viral internalization. A similar case has been made for filovirus entry via macropinocytosis (see below) [18,119]. Therefore, a systematic examination of the relationship between filovirus particle size and the requirement for clathrin‑mediated endocytosis, analogous to the studies with VSV, is warranted.

### 4.2. Evidence for Filovirus Entry via Macropinocytosis

Two studies used the chemical inhibitor EIPA, co-localization with fluid phase markers (high‑molecular-weight dextrans), and dominant-negative Pak-1 (known to inhibit macropinocytosis), to show that EBOV entered Vero cells and HEK-293T cells via a pathway resembling macropinocytosis [17,18]. These studies also showed that inhibition of clathrin, caveolin and DYN-2 did not affect entry of EBOV and morphologically-similar VLPs. Additionally, CtBP1 was shown to be important in this process, presumably as a macropinosome closure factor, although its precise role remains to be determined [17]. Hunt and coworkers [79] showed that infectious EBOV, VLPs and pseudotyped viruses bearing EBOV or MARV GP could use multiple endocytic routes including pathways dependent upon clathrin and resembling macropinocytosis to enter a human glioblastoma cell line and primary human foreskin fibroblasts, Similar results were shown using VLPs in Vero and HeLa cells [118]. Consistent with these findings, Mulherkar and coworkers reported that EBOV enters human peripheral monocyte-derived macrophages and Vero cells through a macropinocytosis-like pathway [119]. Surprisingly, DYN-2 (generally considered dispensable for macropinocytosis [98]) was also found to play an important role in EBOV entry, an observation at odds with two previous reports [17,18]. The basis of these apparent differences in DYN-2 utilization during macropinocytic uptake of viral particles observed among studies using similar viral reagents and cell lines remains unclear and is worthy of further investigation.

The large size and distinctive filamentous morphology of filovirus particles may provide one explanation for why they exploit macropinocytosis-like pathways to enter cells. However, filovirus uptake through macropinocytosis was shown to depend on interactions between components of the viral envelope and cell surface molecules, and not on viral size/morphology per se [18,119]. Recent work suggests that direct interactions between phosphatidylserine (PtdSer) in the outer leaflet of the viral membrane and members of two classes of cell-surface receptors can mediate the macropinocytic uptake of multiple enveloped viruses in tissue culture [125,126]. Specifically, members of the TAM receptor tyrosine kinase family (e.g., Axl, Tyro3) enhance viral uptake and entry via their PtdSer‑binding ligands Gas6 and Protein S, which bridge the virus particle and TAM [125]. PtdSer‑binding members of the TIM family are proposed to enhance viral entry by directly binding to the viral envelope and inducing particle uptake [126]. Both TAM and TIM proteins enhance filovirus entry [127,128,129], and the former do so by stimulating macropinocytic uptake of virus particles [79]. Also using multiple cell lines and primary cells, Axl was shown to facilitate endosomal uptake and membrane fusion of EBOV in a cell type-specific manner. Importantly, Axl did not interact directly with EBOV GP [130]. Whether filoviruses exploit TAM and TIM proteins through PtdSer binding (as seems likely), or through a distinct PtdSer-independent mechanism (see below), remains to be explored. 

Current evidence also points to a role for filovirus GP in inducing uptake through macropinocytosis: VSV pseudotypes bearing EBOV GP induce plasma membrane ruffling upon attachment and are internalized into cells via macropinocytosis; in contrast, VSV pseudotypes bearing VSV G are internalized via clathrin-coated vesicles, as shown previously [18,119]. The simplest hypothesis to explain this observation is that GP interacts with cell-surface receptors that induce macropinocytosis. These putative internalization receptors may include members of the TIM family. The molecular determinants within GP that trigger macropinocytic uptake remain to be defined.

### 4.3. Implications of the Involvement of Multiple Endocytic Pathways in Filovirus Entry

The use of multiple endocytic pathways by filoviruses may influence the outcome of viral infection *in vivo* in several ways, as suggested previously [97,98]. First, viral replication in tissues crucial for the development of filovirus disease might require specific pathways of viral internalization that differ depending upon the cell type. Second, differential use of endocytic pathways by viral isolates, presumably determined by differences in viral interactions with cellular host factors, might contribute to strain-specific patterns of filovirus tissue tropism and virulence. Therefore, future studies dissecting the interactions between filovirus GP (and other components of the viral envelope) and key components of cellular endocytic pathways could prove insightful. 

## 5. Concluding Remarks

**Figure 1:**
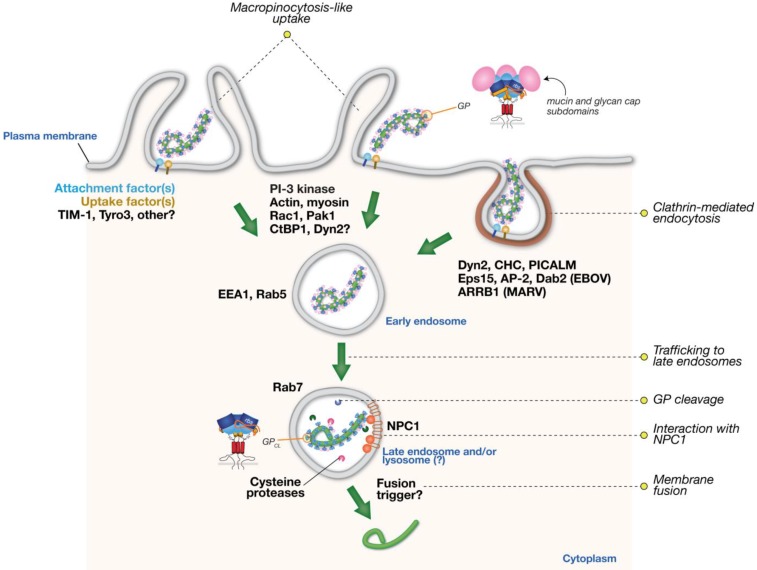
Schematic model of the filovirus entry mechanism with emphasis on clathrin and macropinocytic pathways for viral internalization. Distinct endocytic pathways and host factors implicated in filovirus entry are indicated. Please see the text for a more complete list of identified endocytic host factors and additional details.

Recent work on the cell biology of filovirus entry has advanced our understanding of the endocytic pathways by which these virus particles are internalized into host cells (Figure 1); however, several key questions remain. We believe that future studies should aim to address the following:

- Which cell-surface components trigger viral internalization, and how does their distribution in cells and hosts influence the choice of internalization mechanism (e.g., clathrin *versus* macropinocytosis)?
- How do GP and other viral envelope components (e.g., PtdSer) drive viral internalization and influence the choice of internalization mechanism?
- Are the TIM/TAM PtdSer receptors crucial for viral infection *in vivo* and for pathogenesis?
- How much PtdSer and other anionic lipids are present in the outer membrane leaflet of the filovirus envelope, and how do they get there? Do filoviruses possess a specific mechanism to enhance the levels of these lipids on their outer membrane leaflet?
- Does the pleomorphism of filovirus virions play a role in determining the preference for one internalization pathway over another?
- Are there filovirus strain/species-dependent differences in the mechanism(s) of viral internalization?
- What are the complete sets of host factors required for filovirus entry by clathrin or macropinocytosis-like pathways, and do they differ from the sets of host factors required by other viruses that use similar pathways?
- Are there any overlapping/shared factors between the clathrin and macropinocytosis-like pathways that are involved in filovirus entry? What role (if any) does DYN-2 play in macropinocytic uptake of filoviruses?
- Do the distinct endocytic pathways used by filoviruses converge upon similar downstream compartment(s) from which viral membrane fusion and cytoplasmic escape takes place? Which host pathways mediate delivery of virus particles to these downstream compartments?
- In which intracellular compartment(s) does viral membrane fusion occur?
